# Characterization of Polyelectrolyte Complex Formation Between Anionic and Cationic Poly(amino acids) and Their Potential Applications in pH-Dependent Drug Delivery

**DOI:** 10.3390/molecules22071089

**Published:** 2017-06-30

**Authors:** Zoë Folchman-Wagner, Jennica Zaro, Wei-Chiang Shen

**Affiliations:** 1 Department of Pharmaceutical Sciences, University of Southern California School of Pharmacy, 1985 Zonal Avenue, Los Angeles, CA 90089, USA; folchman@usc.edu; 2 Department of Pharmaceutical Sciences, West Coast University School of Pharmacy, 590 Vermont Ave, Los Angeles, CA 90004, USA; jzaro@westcoastuniversity.edu

**Keywords:** polyelectrolyte complexes, drug delivery, pH-sensitive, histidine

## Abstract

Polyelectrolyte complexes (PECs) are self-assembling nano-sized constructs that offer several advantages over traditional nanoparticle carriers including controllable size, biodegradability, biocompatibility, and lack of toxicity, making them particularly appealing as tools for drug delivery. Here, we discuss potential application of PECs for drug delivery to the slightly acidic tumor microenvironment, a pH in the range of 6.5–7.0. Poly(l-glutamic acid) (E_n_), poly(l-lysine) (K_n_), and a copolymer composed of histidine-glutamic acid repeats ((HE)_n_) were studied for their ability to form PECs, which were analyzed for size, polydispersity, and pH sensitivity. PECs showed concentration dependent size variation at residue lengths of E_51_/K_55_ and E_135_/K_127_, however, no complexes were observed when E_22_ or K_21_ were used, even in combination with the longer chains. (HE)_20_/K_55_ PECs could encapsulate daunomycin, were stable from pH 7.4–6.5, and dissociated completely between pH 6.5–6.0. Conversely, the E_51-dauno_/K_55_ PEC dissociated between pH 4.0 and 3.0. These values for pH-dependent particle dissociation are consistent with the p*K*_a_’s of the ionizable groups in each formulation and indicate that the specific pH-sensitivity of (HE)_20-dauno_/K_55_ PECs is mediated by incorporation of histidine. This response within a pH range that is physiologically relevant to the acidic tumors suggests a potential application of these PECs in pH-dependent drug delivery.

## 1. Introduction

Polyelectrolyte complexes (PECs) are structures that form spontaneously upon combination of oppositely charged macromolecules in solution. The formation and physical characteristics of these complexes is driven by multiple factors including, but not limited to, molecular weight and ionic strength of each component, charge density, concentration, polymer chain rigidity, pH, and mixing intensity [1,2]. The type and concentration of salt in solution is also important as this can result in a neutralization of polymer charge [2,3]. The PECs are formed via an entropic process that has been previously described [4]. Briefly, the complex formation, while largely driven by electrostatics, also involves hydrogen bonding and Van der Waals interactions [1]. The mechanism can be broken into roughly three steps, the first of which is initial complex formation driven by electrostatic interactions, followed by formation of new bonds within this complex, and, lastly, the aggregation of multiple system complexes. The size of PECs is highly controllable, and can be affected by multiple factors including polymer molecular weight and concentration [4]. This property allows for uncomplicated production of PECs within a size range appropriate for drug delivery applications [5].

PECs have several advantages over alternative complexes or nanoparticles in that they have a highly controllable size, avoid toxic cross-linkers, and are biodegradable, biocompatible, and non-toxic [4,6]. Many iterations of PEC work exists in the literature [7,8,9,10,11,12,13], including extensive efforts using chitosan [6,14,15,16,17,18,19], and the complexes have been applied to fields that include drug delivery, gene delivery, and microencapsulation of both cells and tissues [1]. Due to their optimal physicochemical and biological properties, PECs are an ideal area of exploration for development and analysis of potential drug carriers. Owing to the highly controllable formation of PECs, it is of further interest to perform a closer examination of the self-assembly process and properties of PECs formed in a poly(amino-acid) solution. In addition to these advantages, PECs also present a unique opportunity in the potential for modular design enabled by the array of properties inherent to amino acids.

One possibility is to take advantage of the pH-dependent ionization in various amino acid side groups for the design of PECs whose assembly/disassembly properties are pH-sensitive. Previous studies in our lab have demonstrated that complexes between imidazole-modified poly-glutamic acid and polylysine, K_n_, exhibited a response to environmental pH from 5 to 7 on complex formation [20]. Additionally, our lab has previously developed a pH-responsive peptide composed of repeats of glutamic acid and histidine, (HE)_n_ [21]. The pH-responsiveness of this copolymer has been demonstrated at several repeat lengths including (HE)_15_ [22], (HE)_10_ [23], and (HE)_8–12_ [24]. At physiologic pH of ~7.4, glutamic acid is negatively charged, while histidine remains unprotonated, giving (HE)_n_ a net negative charge. As the pH is lowered, the imidazole group in histidine (p*K*_a_ = 6.5) becomes protonated, resulting in neutralization of the (HE)_n_ copolymer. Thus, (HE)_n_ may be used as a highly sensitive pH responsive switch, rapidly converting from charged to uncharged within the small pH window of 6.5–7. Here, we report the use of a longer version of the (HE)_n_ copolymer than previously reported, with 20 repeats it is designated as (HE)_20_.

In this study, we evaluated the combination of pH responsive (HE)_n_ with K_n_ to create a pH-sensitive PEC, capable of forming at neutral pH due to electrostatic interaction of the negative glutamic acid and the positive lysine. At slightly acidic pH, both the histidine (cationic) and glutamic acid (anionic) residues in (HE)_n_ are ionized, resulting in a net-neutral charge of the (HE)_n_ copolymer and loss of electrostatic interaction with lysine. The physicochemical factors necessary for formation of PEC from K_n_ and E_n_ as well as K_n_ and the (HE)_n_ copolymer, including the ultimate pH sensitivity of these PECs, were investigated. The chemotherapeutic drug daunomycin [25] was added to the PEC formed with K_n_ and (HE)_n_ as proof of concept for utility as a drug delivery tool.

## 2. Materials and Methods

### 2.1. Poly(l-glutamic acid)/Poly(l-lysine) PECs

E_n_ and K_n_ were purchased from Alamanda Polymers (Huntsville, AL, USA). The degree of polymerization (DP_n_), as determined by nuclear magnetic resonance spectroscopy, was for E_n_, n = 22 (molecular weight (MW) = 3300) and n = 51 (MW = 7700) and for K_n_, n = 21 (MW = 4400) and n = 55 (MW = 11,500). Aqueous solutions were prepared and filtered through 0.22 µm filters (Argos Technologies, Elgin, IL, USA). Larger molecular weight E_n_ (MW 15,000–50,000) and K_n_ (MW 15,000–30,000) with average DP_n_ of 135 and 127, respectively, were purchased from Sigma Aldrich (St. Louis, MO, USA).

E_n_ and K_n_ were mixed starting at E_n_ concentrations of 1 mg/mL, 0.5 mg/mL, 0.25 mg/mL, or 0.125 mg/mL with K_n_ at 1:1, or 1:2 molar charge in ddH_2_O. PECs were formed by drop-wise addition of E_n_ into a stirred solution of K_n_. E_n_ solution was added in 50 or 100 µL aliquots to 500 µL or 1 mL of K_n_ at 30 s intervals until the full volume of 500 µL or 1 mL was added. The resulting dispersion was stirred for an additional 10 min and then analyzed using dynamic light scattering (DLS) (Dynapro Plate Reader, Wyatt, Santa Barbara, CA, USA) to determine particle size.

Size readings were performed in quadruplicate, where data was shown as average size diameter reading over the four wells ± standard deviation (nm), and outliers were identified using Grubb’s test with α = 0.05. Studies on size and polydispersity of PECs were performed by incubating PEC dispersions at either 25 °C or 4 °C for various periods of time followed by analysis of size variance and polydispersity index (PDI) using DLS.

### 2.2. Recombinant Expression of (HE)_20_ Peptide

The pH-responsive (HE)_20_ peptide was produced recombinantly as a glutathione S-transferase (GST)-(HE)_n_ fusion protein containing a thrombin cutting site between the GST and (HE)_n_ domains as previously described [23]. Briefly, plasmids containing the *GST-(HE)_20_* gene were transformed into *Escherichia coli* expression strain BL21 and the recombinant protein was expressed as previously described [21,23,24]. The resultant bacterial pellets were resuspended in phosphate buffered saline (PBS) (pH 7.4) containing 0.25 mg/mL lysozyme. After incubation for 30 min on ice, phenylmethylsulfonyl fluoride (1 mM) and Triton X-100 (1%, *v*/*v*) were added to lyse the bacteria. The lysate was centrifuged (15,000× *g* for 30 min at 4 °C) and the supernatant was loaded onto a glutathione (GSH) agarose column pre-balanced with PBS. The GSH column was washed with 1% Triton X-100 in PBS followed by PBS, and then thrombin (Sigma) was loaded onto the agarose followed by incubation for 16 h at room-temperature. After thrombin cleavage, the (HE)_20_ peptide was eluted with PBS and purified by nickel-nitriloacetic acid (Ni-NTA) agarose chromatography using 250 mM imidazole, pH 8.0 as the elution buffer [21]. (HE)_20_ peptide was then dialyzed (molecular weight cut-off (MWCO) 3500 Da) against PBS to remove imidazole, and the purified (HE)_20_ peptide was analyzed by SDS-PAGE with Coomassie blue staining to determine purity and confirm size. The final sequence of the peptide was (HE)_10_-F-(HE)_10_, abbreviated as “(HE)_20_”.

### 2.3. Formulation of PEC with Daunomycin, (HE)_20_, and PLys_55_

Daunomycin HCl was purchased from Cayman Chemical (Ann Arbor, MI, USA). PECs with daunomycin, (HE)_20_, and K_55_ were made by combining 1–0.125 mg/mL daunomycin with 1–0.125 mg/mL (HE)_20_ at equal concentrations ((HE)_20-dauno_). The (HE)_20-dauno_ was then added dropwise to a stirred solution of K_55_ at 1:1 (HE)_20-dauno_:K_55_ molar charge ratio. Resulting PECs were analyzed using DLS to determine particle size as well as fluorescence (excitation/emission of 480/590 nm) to determine daunomycin concentration and incorporation efficiency [26]. The encapsulation efficiency was determined by comparing the ratio of fluorescence intensity between free daunomycin to that of the (HE)_20-dauno_/K_55_ PEC.

### 2.4. Formulation of PEC with Daunomycin, (HE)_20-dauno_, and K_55_ in ZnCl_2_·2NH_4_Cl solution

ZnCl_2_, NH_4_Cl, and tricine were purchased from Sigma-Aldrich (St. Louis, MO, USA). ZnCl_2_·2NH_4_Cl was formed by mixing ZnCl_2_ and NH_4_Cl in a 1:2 molar ratio in ddH_2_O, followed by dilution into a solution of 10 mg/mL tricine as chelator. The ZnCl_2_·2NH_4_Cl/tricine solution, at a 1:2 molar ratio to histidine in (HE)_20-dauno_, was then used to dissolve K_55_ (K_55-Zn_). Then, 1 mg/mL daunomycin was added to an equivalent concentration of (HE)_20-dauno_ dissolved in 10 mg/mL tricine and the pH increased to 8.0. The (HE)_20-dauno_ solution was then added dropwise to a stirred solution of K_55-Zn_ in tricine at a 1:1 (HE)_20-dauno_:K_55_ molar charge ratio. The resulting PECs were stirred for an additional 10 min and then analyzed by DLS.

### 2.5. Formulation of PEC with Daunomycin, E_51_, and K_55_ in ZnCl_2_·2NH_4_Cl solution

One milligram per milliliter daunomycin was added to 1 mg/mL E_51_ solution (E_51-dauno_) in ddH_2_O prior to PEC formation. ZnCl_2_ was prepared as described above and used to dissolve K_55_ (K_55-Zn_). The E_51-dauno_ solution was then added dropwise to a stirred solution of K_55-Zn_ in tricine at a 1:1 molar charge ratio. The resulting PECs were stirred for an additional 10 min, diluted into 10 mg/mL Tricine, and then analyzed by DLS.

### 2.6. pH Sensitivity of E_51-dauno_/K_55-Zn_ PEC and (HE)_20-dauno_/K_55-Zn_

To determine the pH sensitivity of the PEC formed from E_51-dauno_/K_55-Zn_ and (HE)_20-dauno_/K_55-Zn_, the PEC were formed in ddH_2_O or 10 mg/mL tricine solutions. The (HE)_20-dauno_/K_55-Zn_ PEC were formed as described above in ddH_2_O and then diluted into 10 mg/mL Tricine. The pH was adjusted using 1 N HCl, and monitored with an Orion PerpHecT ROSS combination pH Micro Electrode from ThermoFischer Scientific (Waltham, MA, USA). PEC were analyzed at intervals of 1 or 0.5 pH units by DLS; PEC degradation was determined by a DLS reading of 0.00 nm or an ‘incomplete’ reading.

## 3. Results and Discussion

### 3.1. PEC Formed from K_n_ and E_n_

**Figure 1:**
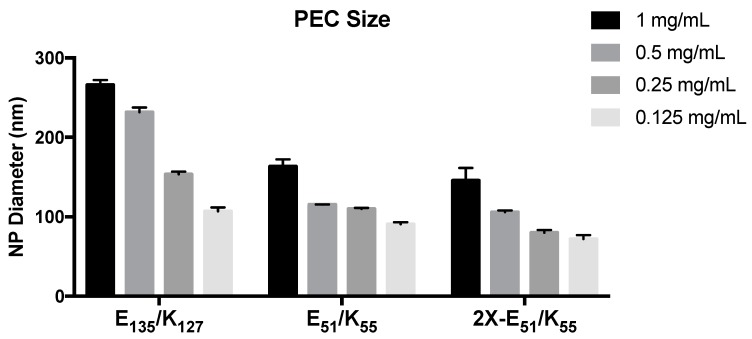
Size summary of poly(l-Lysine) (K_n_) and poly(l-Glutamic Acid) (E_n_) polyelectrolyte complexes (PECs). PECs were formulated at 1:1 or 1:2 molar charge ratios of E_n_:K_n_ with the indicated degree of polymerization (DP_n_) and beginning from an E_n_ concentration of 1 mg/mL. Decreasing the DP_n_ of poly(l-Lysine) and poly(l-Glutamic Acid) from 127/135 to 55/51 resulted in a statistically significant decrease in diameter at all concentrations tested. All formulations demonstrated concentration dependence in diameter. Data shown are hydrodynamic diameter as determined by dynamic light scattering (DLS) and are the averages over 3–4 wells ± standard deviation. Results were analyzed by two-way ANOVA.

PEC showed a high degree of concentration and molecular weight dependence in ultimate hydrodynamic diameter. The PEC formed from E_135_/K_127_ were significantly larger than the E_51_/K_55_ and 2X-E_51_/K_55_ at all concentrations tested (Figure 1). Further, as the concentration of K_n_ and E_n_ decreased, there was a corresponding decrease in the size of the PEC (Figure 1).

This trend was observed for the 1:1 charge ratio of E_135_/K_127_, the 1:1 charge ratio of E_51_/K_55_, and the 1:2 charge ratio of E_51_/K_55_. The concentration dependence of PECs was expected based on well-established models of aggregation by Debye models and Derjaguin, Landau, Verwey, and Overbeek Theory, as well as previous studies [27,28]. PEC formulated with a two-fold excess of positive charge (2X-E_51_/K_55_) were slightly smaller than those formed with an equal charge ratio. This size difference was significant at all concentrations except 0.5 mg/mL. When shorter poly amino-acid chains were used, K_21_ and E_22_, no PEC formation could be seen at a 1:1 charge ratio for E_n_ concentration of 1 mg/mL or 2 mg/mL. When these shorter chains were combined with longer repeats, E_51_/K_21_ and E_22_/K_55_, there remained no complex formation at a 1:1 charge ratio or 1:1 molar ratio. This result suggests that there is a critical limitation for PEC size using these poly amino-acids. As some of the driving factors for PEC formation include molecular weight, charge density, and hydrophobicity, it is reasonable to suggest that the DP_n_ of 21 and 22 did not have sufficient charge density and/or hydrophobicity to nucleate the formation of PEC. The lack of PEC formation even when paired with a long poly amino-acid further supports this claim that there is a bottom limit to PEC formation when working with these short chain amino-acids. The details of this process are as yet unclear, as the process of PEC formation is multi-step. It is well established that increasing concentration of poly-electrolytes in solution decreases the Debye length of the system, resulting in a reduction in electrostatic repulsion [29,30]. It is, therefore, possible that the short chain length of K_21_ and E_22_ did not have enough charge density to decrease the Debye length, resulting in high electrostatic repulsion.

**Figure 2:**
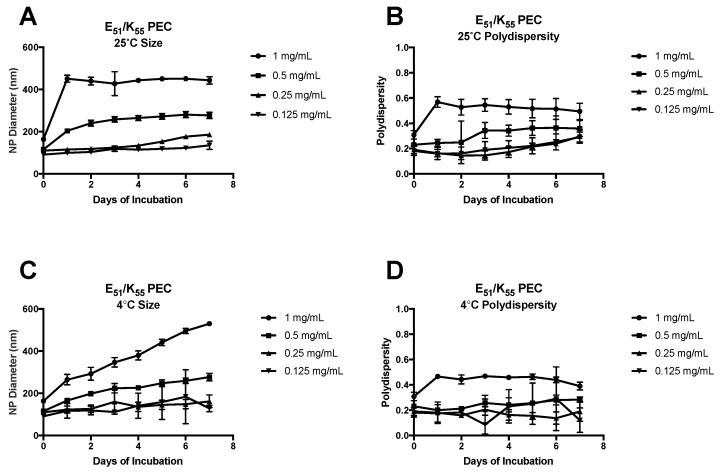
Stability of E_51_/K_55_ PEC formed from 1:1 charge ratio at 25 °C and 4 °C. Panels (**A**,**B**) show the size and polydispersity, respectively, of PEC formed from E_51_ and K_55_ at a 1:1 charge ratio at 25 °C over 7 days. At 1 mg/mL and 0.5 mg/mL there was an increase in size and polydispersity after 24–72 h of incubation, followed by plateau. The 0.25 and 0.125 mg/mL formulations showed little change in diameter over 7 days and a slight increase in polydispersity; Panels (**C**,**D**) show the size and polydispersity, respectively, of PEC formed from E_51_ and K_55_ at a 1:1 charge ratio at 4 °C over 7 days. No plateau was reached in diameter for the 1 mg/mL or 0.5 mg/mL formulations, while the 0.25 and 0.125 mg/mL formulations showed little change. The polydispersity showed an increase after 24 h in the 1 mg/mL formulation, followed by plateau, while the lower concentrations remained nearly constant with minor fluctuations. Data were obtained by DLS, and are displayed as averages over 3–4 wells ± standard deviation.

**Figure 3:**
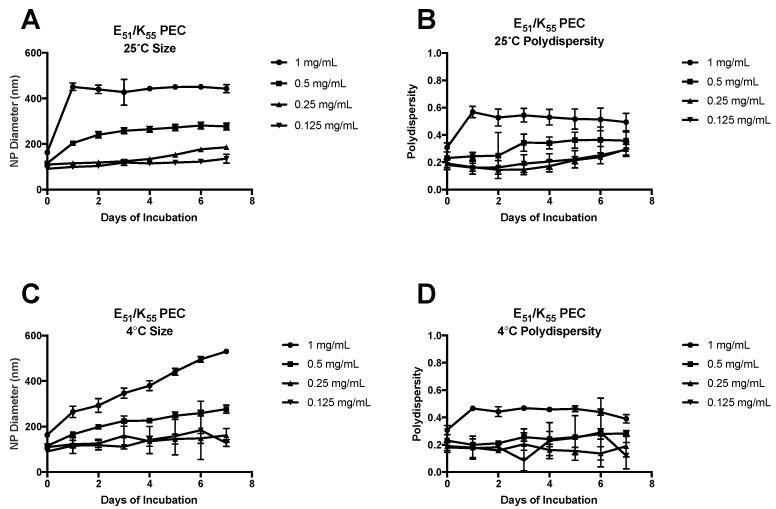
Stability of E_51_/K_55_ PEC formed from 1:2 charge ratio at 25 °C and 4 °C. Panels (**A**,**B**) show the size and polydispersity, respectively, of PEC formed from E_51_ and K_55_ at a 2:1 charge ratio at 25 **°**C over 7 days. At 1 mg/mL and 0.5 mg/mL there was an increase in size after 24 h of incubation, followed by plateau. There was little change in polydispersity. The 0.25 and 0.125 mg/mL formulations showed little change in diameter or polydispersity over 7 days; Panels (**C**,**D**) show the size and polydispersity, respectively, of PEC formed from E_51_ and K_55_ at a 2:1 charge ratio at 4 **°**C over 7 days. All formulations except 0.125 mg/mL showed a gradual increase in size over 7 days. The 0.125 mg/mL formulation showed no significant change in diameter during the 7 day incubation. The 1 and 0.5 mg/mL formulations increased in polydispersity for three days before reaching a plateau, while the 0.25 and 0.125 mg/mL formulations remained stable until day 7, where there was an increase in the polydispersity. Data were obtained by DLS, and are displayed as averages over 3–4 wells ± standard deviation.

The E_51_/K_55_ and 2X-E_51_/K55 were analyzed for size and polydispersity at 25 °C and 4 °C (Figure 2 and Figure 3) at various concentrations from 1 to 0.125 mg/mL E_51_.

At the high end of the concentration range, with E_n_ concentration of 1 mg/mL, the E_51_/K_55_ PEC showed a dramatic increase in diameter after 24 h of 25 °C incubation, nearly doubling in size, and were then constant for 7 days (Figure 2A). The polydispersity of these PEC showed the same initial increase, then proceeded with a downward trend up to the last time-point (Figure 2B). When stored at 4 °C, the E_51_/K_55_ PEC at 1 mg/mL E_51_ had a gradual and sustained increase in size up to the last time point, with no clear plateau reached (Figure 2C). The polydispersity of the 1 mg/mL formulation at 4 °C increased after 24 h and was most stable for the following 6 days (Figure 2D). A similar trend was seen in the PEC formed at an E_51_ concentration of 0.5 mg/mL, however, the size increase in both storage temperatures was more gradual, and no clear size plateau was reached at 4 °C. The polydispersity at 0.5 mg/mL, when stored at 25 °C, showed no change until day 3, where there was an increase from ~0.2 to ~0.35, followed by stabilization of the reading for the remaining 4 days. PEC formed from 0.25 and 0.125 mg/mL were mostly constant in size at both storage conditions. Under a 25°C incubation, the PEC were stable in size for approximately 5 days before increasing slightly. At 4 °C, the PEC remained at a stable size for the full 7 days. The polydispersity was more varied for these lower concentration PECs. The 0.5 mg/mL E_51_ displayed a trend similar to 1 mg/mL of increase followed by plateau, however, the 0.25 and 0.125 mg/mL E_51_ PEC maintained a fairly constant polydispersity but began to show a slight increase after 4 days of incubation at 25 °C. At 4 °C the polydispersity remained constant, however, there was noticeably higher, although not statistically significant, deviation between days.

Similar trends in stability were observed for the PEC formulated with a 2-fold molar charge excess of K_55_ over E_51_ (Figure 3).

With the higher concentration of poly amino-acid solutions (1 mg/mL and 0.5 mg/mL), the complexes stored at 25 °C increased sharply in size after 24 h, and then remained constant for the remaining 7 days (Figure 3A). The polydispersity of these complexes remained consistent for 7 days of 25 °C incubation (Figure 3B). Conversely, for the PEC stored at 4 °C, the complex size increased slowly up to day 3, and remained relatively constant after that (Figure 3C). The polydispersity of the 4 °C PEC was inconsistent with that at 25 °C, showing an increase for 3 days, followed by plateau (Figure 3D). The 0.5 mg/mL E_51_ concentration PEC demonstrated a similar 24 h increase at 25 °C followed by plateau, while the 4 °C PEC increased in size steadily for the 7 days of the study. The polydispersity of the 25 °C formulation remained fairly constant throughout the 7 days, while the polydispersity of the 4 °C formulation increased slowly for 3 days before leveling off. The 0.25 mg/mL concentration formulation showed a very gradual increase in diameter and reached a plateau after approximately 3 days, while the 4 °C formulation continued to show small daily size increases with no plateau. The polydispersity for both formulations was relatively constant, with a sharp increase after 7 days at 4 °C. Lastly, the most dilute of the formulations, 0.125 mg/mL, displayed a fairly constant size for all 7 days of the experiment. Similarly, the 4 °C formulation did not change in size in the dispersion at ~80 nm until day 7. There was no significant change in polydispersity during the 25 °C incubation, while the 4 °C incubation remained constant until day 7, where it increased from ~0.3 to ~0.5.

The plateau reached in almost all cases except the most dilute formulation suggests that there is a most stable form of the E_n_/K_n_ PEC. The last step in PEC formation, as outlined in the introduction, is aggregation of multiple complexes formed by electrostatic interactions [1,4,30]. This is likely a dynamic process that continues until an equilibrium is reached [30]. The lack of plateau in the 4 °C storage is likely due to a decrease in the thermodynamic potential of the system, thus, increasing the time to equilibrium. The PEC formulated with a 2-fold molar excess of K_n_ should have a large positive surface charge, thus, providing electrostatic stability to the system by repulsion between neighboring complexes. In most cases, there is a decrease in time for equilibrium plateau to be reached, which tended to occur after 24 h of incubation.

### 3.2. Formulation of PEC with Daunomycin, (HE)_20_, and K_55_

**Figure 4:**
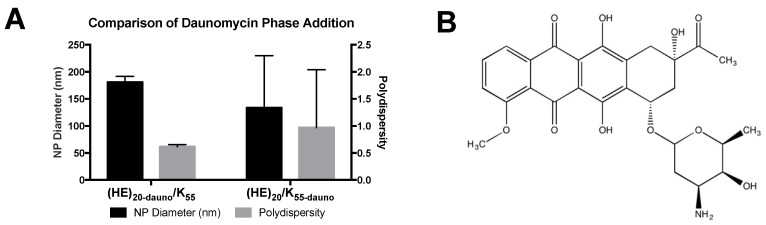
Comparison of daunomycin phase addition. (**A**) In two separate experiments daunomycin was dissolved in the histidine and glutamic acid peptide ((HE)_20_) solution ((HE)_20-dauno_) prior to PEC formation or in the K_55_ solution (K_55-dauno_). The PEC formed from (HE)_20-dauno_ and K_55_ were ~180 ± 10 nm in diameter with a polydispersity index (PDI) of 0.6 ± 0.04. Conversely the PEC formed from (HE)_20_ and K_55-dauno_ were ~130 ± 96 nm in diameter with a PDI of 0.96 ± 1.0. Data shown are averages of 4 wells ± standard deviation; (**B**) Structure of encapsulated daunomycin.

Initial formulation of PEC with E_n_/K_n_ was crucial in establishing both the technique for and limitations of the complex formation. Preliminary experiments utilizing the (HE)_10_ peptide mixed with K_21_ and E_22_ did not form complexes, as expected based on results with E_22_ and K_21_. Therefore, the number of HE-repeats was increased to n = 20 ((HE)_20_). In these complexes, no PEC formation was possible without the addition of daunomycin. Once daunomycin was added into the formulation, the homogeneity of the resulting PEC was dependent on whether daunomycin was added to the polyanion ((HE)_20_) or polycation (K_55_) phase (Figure 4).

When daunomycin was combined with (HE)_20_ prior to PEC formation ((HE)_20-dauno_/K_55_), the resulting complexes were approximately 180 nm with a PDI of 0.60. There was, additionally, low deviation between the samples tested. Conversely, when daunomycin was added to the polycation prior to PEC formation ((HE)_20_/K_55-dauno_), the resulting complexes were ~133 nm in diameter with a PDI of nearly 1. Additionally, there was large variation between samples for the PEC formed from (HE)_20_/K_55-dauno_, with standard deviation of ~100 nm for PEC diameter and ~1.0 for PDI (Figure 4).

These results suggest that the incorporation of daunomycin into the PEC is driven by both electrostatic and hydrophobic interactions. At pH values above the p*K*_a_ of histidine (6.5), the (HE)_20_ copolymer will have a net negative charge and is, therefore, capable of electrostatic interaction between the positively charged daunomycin (p*K*_a_ 8.4 [31]) and the gamma-carboxyl group of glutamic acid in (HE)_20_. Further interaction may be possible due to hydrophobic interactions between the hydrophobic daunomycin (logP 1.8 [32]) and the histidine residues in the (HE)_20_ copolymer. The polycationic K_55_ lacks the potential for electrostatic interaction with daunomycin, and the higher charge density of the lysines would likely repel interactions with the drug. The lack of discrete PEC formation upon addition of daunomycin to the polycation K_55_ phase supports the probability that the driving force for encapsulation of daunomycin in the PECs is electrostatic and hydrophobic interactions between daunomycin and (HE)_20_, which form prior to the addition of the polyanion phase and, thus, result in drug incorporation once the (HE)_20_/K_55_ PECs are formed. Based on the heterogeneity of the (HE)_20_/K_55-dauno_ PEC, the daunomycin was exclusively added to the polyanion phase, (HE)_20_, in all further formulations.

**Figure 5:**
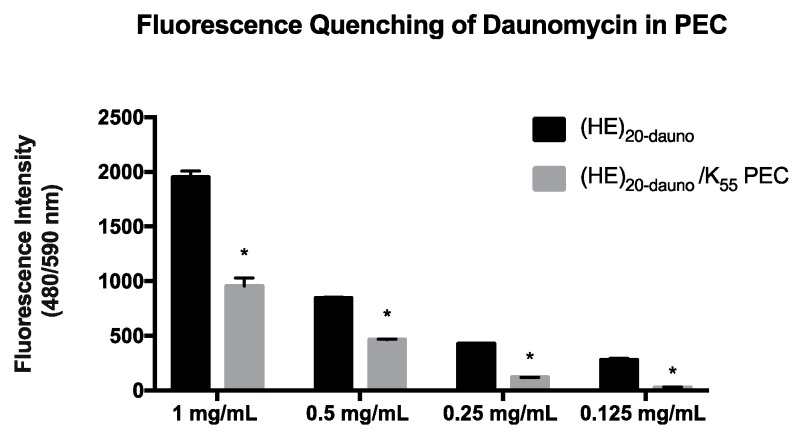
Fluorescence quenching of daunomycin in (HE)_20-dauno_/K_55_ PEC. Fluorescence measurements were taken of the (HE)_20-dauno_ solution alone and the (HE)_20-dauno_/K_55_ PEC. There is a significant decrease in fluorescence signal from daunomycin at all concentrations of PEC. The data shown are averages over 4 wells ± standard deviation. Results were analyzed by the Student’s *t*-test, with *p* < 0.001 indicated (*).

PEC formed from (HE)_20-dauno_/K_55_ showed a decrease in the fluorescent signal of daunomycin (Figure 5) at all concentrations tested. The fluorescence quenching observed in Figure 5 was attributed to encapsulation of daunomycin within the PEC, thus dampening the fluorescent signal [33]. The fluorescence intensity of free daunomycin was compared to the fluorescent signal of the (HE)_20-dauno_/K_55_ PEC, and the decrease in signal was taken as proportional to encapsulation efficiency. The loading amount (wt %) of daunomycin in PEC solutions with concentrations of 1–0.125 mg/mL polyanion and an equal molar charge of polycation, was 36% at all formulations. The encapsulation efficiency ranged from 58% to 85% (Table 1).

**Figure 6:**
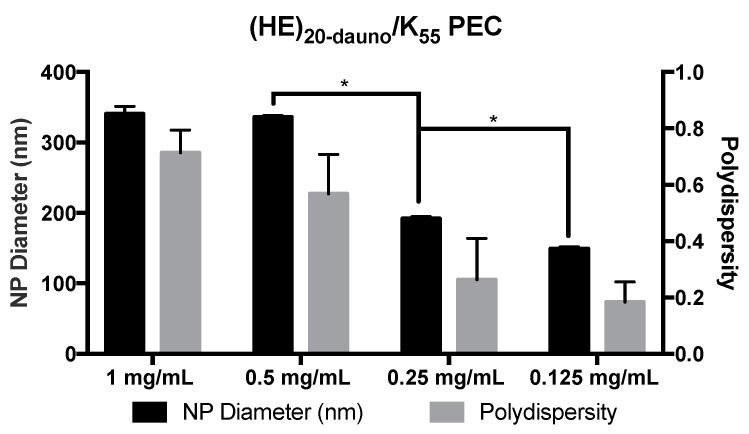
PEC formulated from (HE)_20-dauno_ and K_55_ PEC formulated from (HE)_20-dauno_/K_55_ demonstrated significant concentration dependence in size at concentrations below 1 mg/mL. Polydispersity of the PEC decreased along with the size, and was statistically significant at concentrations below 1 mg/mL. Data shown are averages of four wells ± standard deviation. Results were analyzed by two-way ANOVA with *p* < 0.05 indicated (*).

The (HE)_20-dauno_/K_55_ complexes also demonstrated a concentration dependent size decrease, with average diameter and polydispersity decreasing as the concentration prior to formulation decreased (Figure 6). These PEC further demonstrate the degree of control available for the ultimate size of the complexes formed.

### 3.3. Formulation of PEC with Daunomycin, (HE)_20_, and K_55_ in ZnCl_2_·2NH_4_Cl Solution

**Figure 7:**
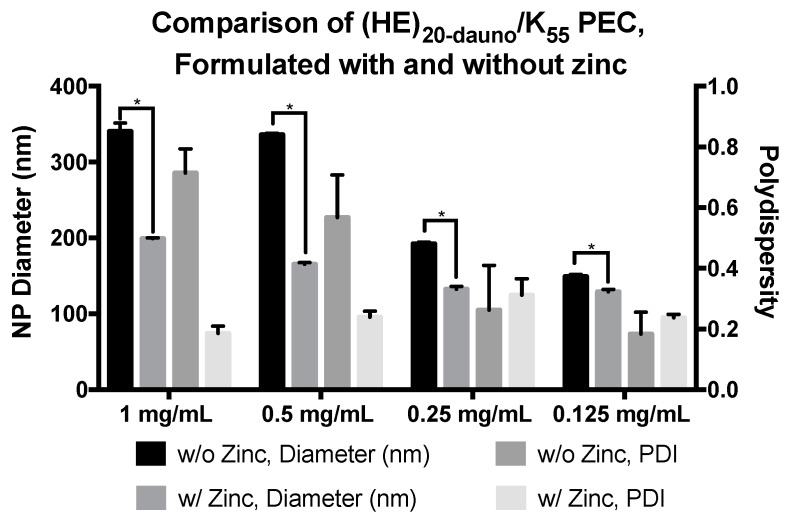
Comparison of PEC formulated from (HE)_20-dauno_ and K_55_ with and without zinc. PEC formulated from (HE)_20-dauno_/K_55_ with zinc were significantly smaller at all concentrations tested than those without zinc. There was a corresponding decrease in polydispersity that was most apparent at the 1 mg/mL and 0.5 mg/mL formulations, however, it was not statistically significant. Data shown are averages of four wells ± standard deviation. Results were analyzed by two-way ANOVA with *p* < 0.0001 indicated (*).

While the PEC did form between (HE)_20-dauno_ and K_55_, the resulting formulations were of varying size with high polydispersity (Figure 6). To mitigate this problem, studies were performed with the addition of zinc to stabilize the PEC through metal ion coordination [34,35,36], thus serving as a stabilizer for the PEC. Histidine is able to coordinate divalent metal ions, including zinc, with high affinity through two to six histidine residues [37]. Additionally, there is evidence for doxorubicin interaction with zinc [38], specifically through the two keto-phenolate substituents [39], which are maintained in daunomycin. The ability of zinc to interact strongly with not only histidine but carbonyl residues is well documented [39,40,41] and, thus, provides the chemical basis for interactions between the zinc ions, the (HE)_20_ copolymer, and encapsulated daunomycin. Therefore, zinc was added to the polycation phase to increase the number of interactions between the (HE)_20-dauno_ and K_55_ to include electrostatic, hydrophobic, and metal ion coordination. Zinc was added to solution in the form of zinc ammonium chloride and dissolved in 10 mg/mL tricine, which was used as a chelator. The zinc ammonium chloride and tricine solution was used to dissolve K_55_, followed by PEC formation as described above. These complexes were significantly smaller in size and had far lower polydispersity (Figure 7) illustrating the effect of the zinc ions in providing additional interaction potential between the (HE)_20-dauno_ and K_55_. The (HE)_20-dauno_/K_55-Zn_ complexes continued to exhibit a concentration dependent size (Figure 7).

### 3.4. pH Sensitivity of E_51-dauno_/K_55-Zn_ PEC and (HE)_20-dauno_/K_55-Zn_

**Figure 8:**
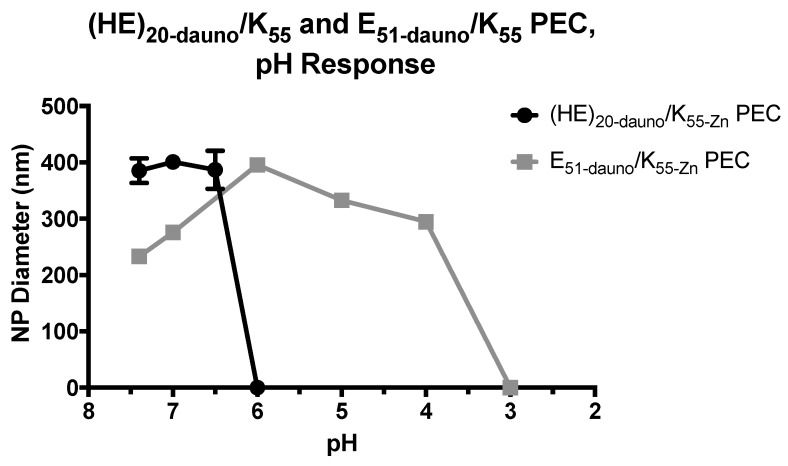
pH dependence of (HE)_20-dauno_/K_55-Zn_ PEC and E_51-dauno_/K_55-Zn_ PEC formulated with (HE)_20_ demonstrate a high degree of pH sensitivity, with complete complex degradation between pH 6.5–6.0. Complex size stayed constant until pH 6.0. PEC formulated from E_51_ and K_55_ showed high variability in complex size over the pH range tested and did not degrade until between pH 4.0–3.0. These data show that the pH sensitivity exhibited by the (HE)_20-dauno_/K_55-Zn_ PEC is specifically mediated by the histidine present in (HE)_20_. Data shown are averages of 3–4 wells ± standard deviation.

The (HE)_20-dauno_/K_55-Zn_ PEC exhibited a high degree of pH sensitivity, dissociating completely at a pH between 6.0 and 6.5 (Figure 8). The complexes were stable in size at pH values of 7.4, 7.0 and 6.5, with little variation in diameter or increase in deviation. The PEC formulated with E_51-dauno_/K_55-Zn_ showed a high degree of variation in size from pH 7.4–4.0, and dissociated completely between pH 3.0 and 4.0 (Figure 8). These results indicate that the pH dependence of the (HE)_20-dauno_/K_55-Zn_ PEC is specifically mediated by the histidine present in (HE)_20_. (HE)_20_, as described above, has a p*K*_a_ of 6.5 for the imidazole group in histidine. At pH 6.5, there will be a 1:1 ratio of ionized to unionized histidine residues. The PEC formation is dependent on charge interaction between the negative glutamic acid of (HE)_20_ and the positive lysine of K_55_. When histidine in (HE)_20_ begins to protonate, the overall charge of the (HE)_20_ copolymer is lost, resulting in loss of the electrostatic interactions that held together the PEC. This result was also seen in the E_51-dauno_/K_55-Zn_, which dissociates at a pH of 3.0, a value below the p*K*_a_ of the gamma-carboxyl group of glutamic acid in E_51_. The loss of charge on the (HE)_20_ copolymer at pH 6.0–6.5 will additionally result in loss of the electrostatic interactions present in the (HE)_20_-daunomycin interaction discussed previously. Therefore, it is likely that loss of the PEC will also result in a release of daunomycin from interaction with (HE)_20_ and the accumulation of free drug in solution.

## 4. Summary

This paper outlines the formulation of polyelectrolyte complexes composed of cationic and anionic poly(amino acids). Formulations of PEC with poly(glutamic-acid) and poly(lysine) showed concentration dependent size variation at lengths of E_51_/K_55_ and E_135_/K_137_, however, no PECs were observed when E_22_ or K_21_ were used, even in combination with longer chain-length E_51_ or K_55_ at charge ratios of 1:1, or 1:2. This result suggests a size limitation in PEC formation with these poly(amino acids). While the specific formation process with these poly(amino acids) has yet to be elucidated, it is possible that the short chain length of K_21_ and E_22_ did not have enough charge density to decrease the Debye length, resulting in high electrostatic repulsion and a lack of successful PEC. Complexes formed with E_51_/K_55_ and a two-fold positive charge excess, 2X-E_51_/K_55_, were shown to exhibit a fairly rapid increase in size after 24 h followed by plateau. The change in size was most dramatic at the highest concentrations studied (1 mg/mL and 0.5 mg/mL), with a much smaller size increase at lower concentrations.

To investigate the potential pH sensitivity of these PECs, a (HE)_20_ copolymer composed of repeats of histidine and poly(glutamic acid) was used in place of E_n_ for complex formation. Histidine has previously shown potential as a tool for pH-sensitive drug delivery [42,43,44,45,46,47] and the (HE)_20_ copolymer specifically has been shown to enable pH-dependent internalization of cell penetrating peptides [21,23], thus making it a promising candidate for formation of pH-sensitive PECs. The (HE)_20_ copolymer was mixed with K_55_ at 1:1 molar charge ratios and, in the presence of zinc, was able to encapsulate the anticancer drug daunomycin. These (HE)_20-dauno_/K_55-Zn_ PEC showed a high degree of pH responsiveness, maintaining a ~400 nm diameter at pH values of 7.4–6.5, and dissociating completely between pH 6.5 and 6.0. In contrast, PEC formed with E_51-dauno_/K_55-Zn_ did not dissociate until between pH 4.0 and 3.0. This large difference in pH response is likely mediated specifically by the incorporation of histidine in the (HE)_20_ copolymer. (HE)_20_ will have a net negative charge at pH values above ~ 6.5, however, histidine has a p*K*_a_ of 6.5 and so at a pH of 6.5 will be 50% ionized. This ionization partially neutralizes the negative charge of (HE)_20_, and as the copolymer loses its net negative charge, the electrostatic interactions with K_n_ driving the PEC formation are lost, and the particle dissociates. The E_51-dauno_/K_55-Zn_ particles, lacking histidine, do not dissociate until a pH between pH 4.0 and 3.0, which is close to the p*K*_a_ of poly(glutamic acid). At pH 3.0 the poly(glutamic acid) residues start to become protonated, thus losing the charge-based interaction with K_n_ and falling apart.

The use of pH-responsive platforms for drug delivery has garnered increased attention due to the fact that solid tumors possess a slightly acidic environment (pH 6.5–7) compared to that of healthy physiologic fluid (pH 7.4) [48,49,50,51,52,53]. This enables the use of stimulus-sensitive carriers, such as the (HE)_20-dauno_/K_55-Zn_ PEC, that respond to the change in pH found in the tumor microenvironment by dissociation of the carrier complex. Further studies in the release profiles of these pH-responsive PECs may illustrate the use of these complexes in enabling site-specific drug release directly to the tumor microenvironment, ultimately resulting in higher specificity of drug action, lower side-effects, and reduced dosage. The (HE)_20-dauno_/K_55-Zn_ PEC demonstrate a controllable size and pH response and offer a highly modular platform from which to continue further studies into the use of this technology for pH-dependent drug delivery to the tumor microenvironment.

## 5. Conclusions

This paper outlines the production of polyelectrolyte complex nanoparticles formulated from poly(l-glutamic acid) and poly(l-lysine) at low concentrations from 1 to 0.125 mg/mL. The complexes formed are highly controllable in size, and a critical length limitation of >~50 repeats was noted for successful complex formation. The addition of histidine to these complexes, in the form of the copolymer (HE)_20_, resulted in polyelectrolyte nanoparticles that retained a high dependence on concentration for ultimate size. Additionally, the (HE)_20_/K_55_ nanoparticles, with encapsulated daunomycin and zinc, were highly pH-responsive, degrading completely between pH 6.5–6.0, demonstrating the effectiveness of histidine in conferring pH-sensitivity.

## Figures and Tables

**Table 1:** Encapsulation efficiency and loading content of daunomycin in (HE)_20-dauno_/K_55_ PEC. Fluorescence measurements were taken of the daunomycin solution alone and the (HE)_20-dauno_/K_55_ PEC. The ratio between free drug and drug encapsulated within the PEC was used to determine the encapsulation efficiency and loading content.

Daunomycin Concentration (mg/mL)	Encapsulation Efficiency, %	Loading Content, wt %
1	68%	25%
0.5	58%	21%
0.25	74%	27%
0.125	85%	31%
